# Gait rehabilitation for foot and ankle impairments in early rheumatoid arthritis: a feasibility study of a new gait rehabilitation programme (GREAT Strides)

**DOI:** 10.1186/s40814-022-01061-9

**Published:** 2022-05-30

**Authors:** Gordon J. Hendry, Lindsay Bearne, Nadine E. Foster, Emma Godfrey, Samantha Hider, Lisa Jolly, Helen Mason, Alex McConnachie, Iain B. McInnes, Aimie Patience, Catherine Sackley, Mandeep Sekhon, Bethany Stanley, Marike van der Leeden, Anita E. Williams, Jim Woodburn, Martijn P. M. Steultjens

**Affiliations:** 1 School of Health and Life Sciences, Glasgow Caledonian University, Glasgow, UK; 2 Department of Population Health Sciences, King’s College London, London, UK; 3 Centre for Applied Health and Social Care Research, Kingston University and St George’s, University of London, London, UK; 4 School of Medicine, Keele University, Newcastle, UK; 5 STARS Education Alliance, Surgical Treatment and Rehabilitation Service (STARS), The University of Queensland and Metro North Health, Herston, Queensland Australia; 6 Midlands Partnership Foundation Trust, Stafford, UK; 7 Clinical Research and Development, NHS Greater Glasgow and Clyde, Glasgow, UK; 8 Yunus Centre for Social Business and Health, Glasgow Caledonian University, Glasgow, UK; 9 Robertson Centre for Biostatistics, Institute of Health and Wellbeing, University of Glasgow, Glasgow, UK; 10 Institute of Infection, Immunity and Inflammation, University of Glasgow, Glasgow, UK; 11 School of Health Sciences, University of Nottingham, Nottingham, UK; 12 Amsterdam University Medical Centre/Reade, Amsterdam, The Netherlands; 13 School of Health and Society, University of Salford, Salford, UK; 14 School of Health Sciences and Social Work, Griffith University, Gold Coast, Queensland Australia

**Keywords:** Gait rehabilitation, Rheumatoid arthritis, Feasibility

## Abstract

**Background:**

Foot impairments in early rheumatoid arthritis are common and lead to progressive deterioration of lower limb function. A gait rehabilitation programme underpinned by psychological techniques to improve adherence, may preserve gait and lower limb function. This study evaluated the feasibility of a novel gait rehabilitation intervention (GREAT Strides) and a future trial.

**Methods:**

This was a mixed methods feasibility study with embedded qualitative components. People with early (< 2 years) rheumatoid arthritis (RA) and foot pain were eligible. Intervention acceptability was evaluated using a questionnaire. Adherence was evaluated using the Exercise Adherence Rating Scale (EARS). Safety was monitored using case report forms. Participants and therapists were interviewed to explore intervention acceptability. Deductive thematic analysis was applied using the Theoretical Framework of Acceptability. For fidelity, audio recordings of interventions sessions were assessed using the Motivational Interviewing Treatment Integrity (MITI) scale. Measurement properties of four candidate primary outcomes, rates of recruitment, attrition, and data completeness were evaluated.

**Results:**

Thirty-five participants (68.6% female) with median age (inter-quartile range [IQR]) 60.1 [49.4–68.4] years and disease duration 9.1 [4.0–16.2] months), were recruited and 23 (65.7%) completed 12-week follow-up. Intervention acceptability was excellent; 21/23 were confident that it could help and would recommend it; 22/23 indicated it made sense to them. Adherence was good, with a median [IQR] EARS score of 17/24 [12.5–22.5]. One serious adverse event that was unrelated to the study was reported. Twelve participants’ and 9 therapists’ interviews confirmed intervention acceptability, identified perceptions of benefit, but also highlighted some barriers to completion. Mean MITI scores for relational (4.38) and technical (4.19) aspects of motivational interviewing demonstrated good fidelity. The Foot Function Index disability subscale performed best in terms of theoretical consistency and was deemed most practical.

**Conclusion:**

GREAT Strides was viewed as acceptable by patients and therapists, and we observed high intervention fidelity, good patient adherence, and no safety concerns. A future trial to test the additional benefit of GREAT Strides to usual care will benefit from amended eligibility criteria, refinement of the intervention and strategies to ensure higher follow-up rates. The Foot Function Index disability subscale was identified as the primary outcome for the future trial.

**Trial registration:**

ISRCTN14277030

**Supplementary Information:**

The online version contains supplementary material available at 10.1186/s40814-022-01061-9.

## Key messages regarding feasibility

- What uncertainties existed regarding the feasibility?

Prior to evaluation via a future randomised controlled trial, we wanted to establish whether the newly developed GREAT Strides gait rehabilitation programme was likely to be safe and acceptable to people with early RA. We also sought to establish whether GREAT Strides would be acceptable to therapists (podiatrists and physiotherapists) responsible for its delivery, and whether it could be delivered as intended. Prior to evaluation in a randomised trial, uncertainty about the best and most practical outcome measure in terms of ability to detect change for use as primary outcome measure. Uncertainty also existed concerning the feasibility of a main trial in terms of recruitment and retention rates relative to sample size requirements.

- What are the key feasibility findings?

We identified that GREAT Strides was likely to be safe, had excellent patient acceptability, and resulted in good patient adherence. GREAT Strides was acceptable to intervention therapists and was delivered with high fidelity. Recommendations are provided for primary outcome measure selection for a future main trial (the Foot Function Index Disability subscale). Recruitment and retention rates were lower than anticipated and require further strategies in a future main trial.

- What are the implications of the feasibility findings for the design of the main study?

Results from evaluation of patient acceptability and adherence are promising and warrant further evaluation of the added benefit of GREAT Strides to usual care in a randomised trial. Evaluations of patient and therapist acceptability and fidelity have informed important refinements of the GREAT Strides intervention and therapists’ intervention training format and resources. The Foot Function Index disability subscale is recommended as the primary outcome for the future randomised trial on the basis of it appearing to be the most theoretically consistent and its practical characteristics. Recruitment and retention rates observed necessitated refinement of eligibility criteria, follow-up procedures, and data collection methods and will be tested in a pilot randomised trial.

## Background

Approximately 645,000 people have rheumatoid arthritis (RA) in the UK and most will experience foot and mobility problems [1–3]. In early disease around 60–65% of patients experience foot pain and joint swelling, and walking-related disability which may be persistent and progressive [4]. People with RA often exhibit slow and unsteady gait characterised by decreased walking speed, ankle power, and step length [5–7]. They take fewer steps and are more sedentary than healthy adults [8–11]. These sedentary characteristics are associated with poor body composition and elevated cardiovascular disease risk [12–14]. Deteriorations in gait characterised by lower limb muscle weakness, poor muscle endurance, and reduced proprioception/balance are common and are associated with impaired physical function and difficulties with activities of daily living [6, 7, 15–22]. Self-reported walking disability at 2 years post-diagnosis has been identified as the main predictor of persistent walking disability [3]. This suggests that there may be a therapeutic ‘window of opportunity’ for preservation of gait and prevention of persistent walking disability during early disease.

Exercise is a key treatment for people with RA but adherence to exercise tends to be poor. This may be due to concerns about safety, lack of knowledge or skills or individuals’ beliefs about exercise [23–26]. These concerns may be exacerbated in people with depression/anxiety or poor exercise self-efficacy [23, 27–29]. To address these concerns, accurate information about exercise and strategies to enhance motivation and exercise self-efficacy are needed. Targeting these factors in a theoretically informed behaviour change intervention is recommended [30], as there is strong evidence that weight-bearing exercises and physical activity are safe and do not cause disease exacerbations or joint damage [10].

Gait rehabilitation that includes repetitive practice of gait cycles is an evidence based treatment to improve independent walking capacity in neurological disorders [31–36]. Two studies have demonstrated benefits in walking capacity in people with RA from rehabilitation programmes which included repetitive walking tasks [37, 38]. Whilst walking is generally promoted as a healthy behaviour, gait rehabilitation is not included in clinical guidelines nor provided as part of usual care for people with early RA. We developed and investigated the feasibility and acceptability of a novel psychologically informed gait rehabilitation intervention for adults with early RA (GREAT Strides) and investigated key parameters for assessment in a future randomised controlled trial.

## Methods/design

### Aims and objectives

The study objectives were to (i) evaluate feasibility and acceptability of GREAT-strides; and (ii) the feasibility of a future trial. More specifically, aims were

1. To evaluate patient and therapists’ perceptions of acceptability of the gait rehabilitation intervention.
2. To evaluate the initial safety of, and adherence to the gait rehabilitation intervention.
3. To evaluate the fidelity of intervention therapist training and delivery of the gait rehabilitation intervention.
4. To evaluate selected measurement properties and characteristics of candidate outcome measures to select the most suitable primary outcome measure for a future main trial.
5. To monitor rates of recruitment, attrition and data completeness.

### Design

This study was a multi-centre (*n* = 4), single-arm, repeated measures (pre- and post-intervention) design with nested qualitative interviews. This feasibility study was reviewed and approved by the West of Scotland Research Ethics Committee 3 (17/WS/0264) in January 2017.

### Settings

The study was conducted in outpatient rheumatology (recruitment) and rehabilitation (physiotherapy and podiatry) settings in 4 United Kingdom National Health Service (NHS) Hospitals in Glasgow (Gartnavel General Hospital and Glasgow Royal Infirmary, NHS Greater Glasgow and Clyde), Stoke-on-Trent (Haywood Hospital, Midlands Partnership Foundation Trust), and London (King’s College Hospital NHS Foundations Trust).

### Participants

Participants were included if they (a) were aged ≥ 18 years, (b) diagnosed with RA (American College of Rheumatology (ACR) 2010 classification criteria [39]) within the previous 2 years, (c) had disease-related foot impairments defined as (i) self-reported foot pain, and/or (ii) the presence of foot/ankle joint synovitis/tenosynovitis detected during routine rheumatology clinical examination. Exclusion criteria included contraindications to the intervention identified by their consulting rheumatologist, those who were unable or unwilling to provide informed consent; or were taking part in other non-medical intervention studies affecting lower limb function.

### Recruitment

Potential participants were identified by rheumatology team members in one of two ways. Rheumatology clinic lists were screened by the direct care team and potentially eligible patients who were interested in the study were introduced to the research team. A second approach involved initial screening of clinic lists by trial personnel at participating sites. Written invitation letters were sent to potentially eligible patients. Patients interested in the study responded using an expression of interest form and were contacted by the recruiting researcher to confirm eligibility and willingness to participate. Once eligibility was confirmed, all eligible patients were invited to attend an initial appointment where written informed consent was obtained and baseline assessment completed.

### Intervention development

Informed by MRC guidelines for the development of complex interventions [40, 41], GREAT Strides was developed by people with RA, rheumatology specialist physiotherapists, podiatrists, health psychologists, and clinical academics experienced in intervention development. It drew on existing exercise programmes for older adults and people with RA [37, 38, 42–45], and incorporated psychological components to address adherence [46–49]. A series of patient and public involvement (PPI) and stakeholder engagement workshops/interviews were conducted. At the final workshop, the specific intervention components and therapist training plans were agreed by consensus by the trial management group.

### The GREAT Strides intervention

GREAT Strides is a theoretically underpinned psychologically informed home-based 12-week gait rehabilitation programme which included two compulsory face-to-face sessions and up to four additional sessions with trained therapists over a 12-week period. GREAT Strides was comprised of

**Fig. 1:**
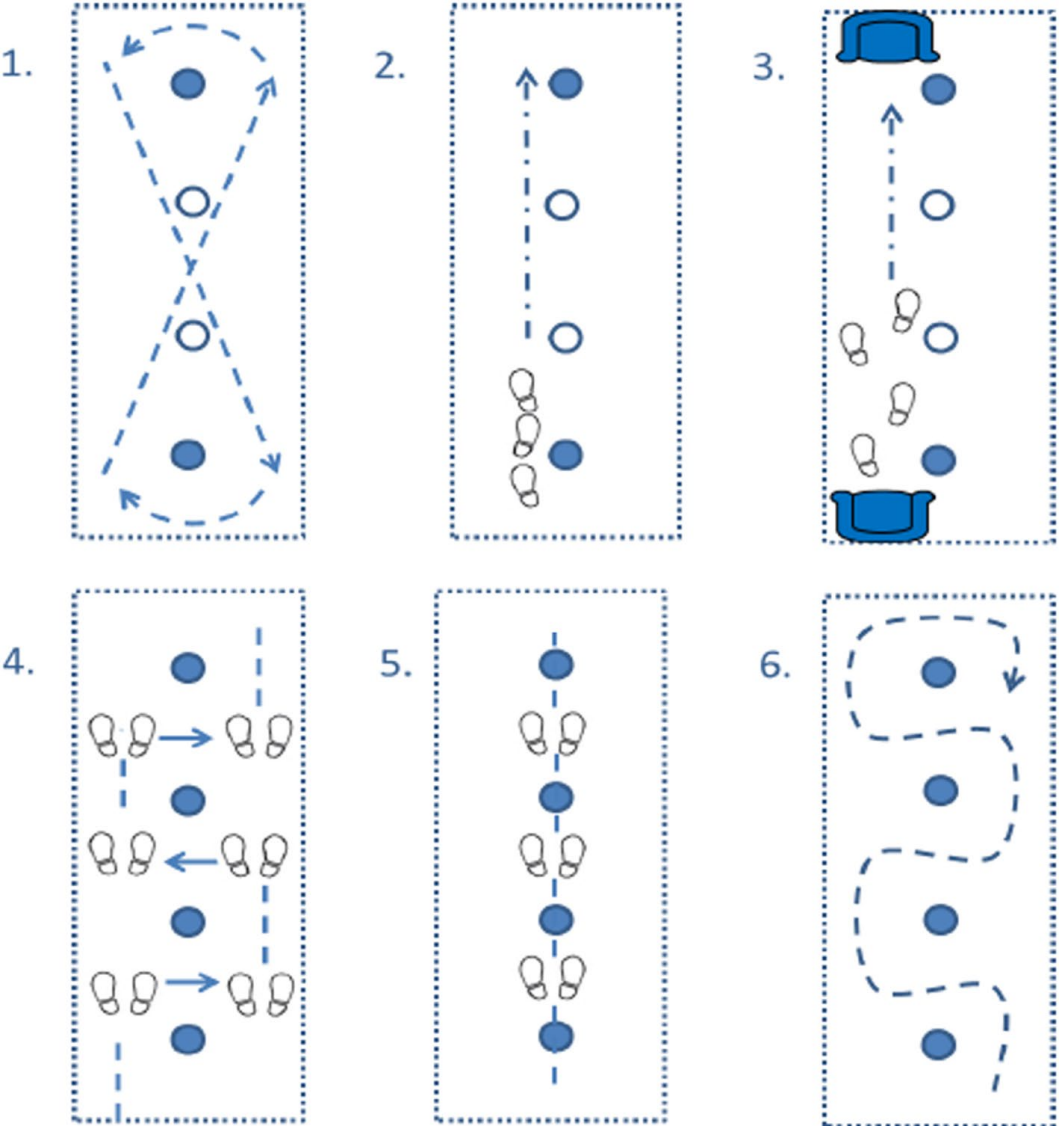
GREAT Strides gait rehabilitation circuit. **1** Figure of 8 walk. **2** Heel-to-toe walk. **3** Get up and go. **4** Obstacle side-step. **5** Obstacle step-over. **6** Obstacle walk-around

- A circuit of six repetitive walking tasks designed for setup and completion at home (Fig. 1).
- The psychological components of GREAT Strides are based upon motivational interviewing (MI) to support participants to overcome barriers and facilitate translation of intentions into action [46, 47]. MI is a collaborative, goal-oriented style of communication where particular attention is paid to the language of change [47]. GREAT Strides is underpinned by the Theory of Planned Behaviour (TBP) [48, 49]. This model places emphasis on individuals’ perceived ability to perform a given behaviour and their attitudes to initiating behavioural change [50]. Specific behaviour change techniques (BCTs) have been incorporated into the intervention to facilitate adherence (Table 1).
- Participants were provided with an educational booklet, an exercise diary, and a digital versatile disc (DVD) including step-by-step demonstrations of gait circuit home set-up and task completion. All resources were also available on a dedicated study website [51].

**Table 1:** The core components and BCTs for GREAT Strides intervention sessions

Session 1		Sessions 2–4	
Core components	BCTs	Core components	BCTs
Gives a short overview of the GREAT intervention. Conducts a brief clinical assessment. Conducts a psychological assessment using the principles of motivational interviewing. Completes the worksheets from the patient support booklet. Gives patient a DVD and manual. Confirms appointment for session 2.	Provides information about health consequences. Verbal persuasion about capability. Discuss discrepancy between current behaviour and goals. Discuss pros and cons. Demonstration of the behaviour. Instructions on how to perform the behaviour. Behaviour practice/rehearsal. Feedback on behaviour. Goal setting (behaviour). Goal setting (outcome). Problem solving. Action planning. Graded tasks. Prompts and cues. Self-monitoring of behaviour. Social support. Commitment.	Review progress on gait circuit. Checks gait circuit progression. Completes and/or reviews the worksheets from the patient support booklet. Signposts to local walking groups.	Verbal persuasion about capability. Review behavioural goal. Feedback on behaviour. Problem solving. Goal setting (outcome). Social support (unspecified). Demonstration of the behaviour. Instructions on how to perform the behaviour. Behaviour practice/rehearsal. Goal setting (behaviour). Graded tasks. Action planning.

Session 1 involves six core components: an overview of the intervention, brief clinical assessment of disease activity and functional status, conducing a psychological assessment using the principles of motivational interviewing, demonstration of the circuit, dose setting, and the delivery of 17 BCTs (Table 1), and provision of support materials. Session 2 (1–4 weeks after session 1) involved delivery of 4 core components and 12 BCTs (Table 1), further dose and circuit adaptation as required. Sessions 3–6 were optional (same core components and BCTs as session 2) and could be face-to-face or telephone-based according to participants’ needs to maintain contact, promote adherence, and/or to provide specific advice regarding progression.

The setting of the starting dose is undertaken by instructing participants to complete one full set of the circuit (1 min per task) followed by immediate rating of perceived exertion using a modified version of the Borg Rating of Perceived Exertion (RPE) scale (range 6–20). The starting dose equals the number of sets required to achieve an RPE from 13 up to 17 (equivalent to 50–80% maximal exertion) [52, 53] and frequency set initially at 3 times per week.

### Therapist intervention training

Eleven therapists across the three sites participated in a bespoke training package (8 h training over 2 days delivered face-to-face 2 weeks apart) to deliver GREAT Strides. Training was delivered by the GREAT trial team (GJH, AP, LB, EG, MS). It included set up and delivery of the gait circuit, dose setting, and how to apply MI and BCTs to help patients complete their walking exercises regularly. Therapists were also provided with a clinician manual to accompany the training content and access to electronic resources on the study website [51].

### Feasibility outcomes

#### Intervention acceptability, safety, and adherence

Participant acceptability was evaluated using a 3-item questionnaire which utilises 5-point Likert scales for responses, adapted from previous trials [54, 55]. Study and intervention safety were monitored by examining rates of serious adverse events (SAEs) and expected events of interest (see Additional file 1) deemed to be related to the intervention. Treatment adherence was evaluated using the Exercise Adherence Rating Scale (EARS), a valid and reliable self-reported measure of adherence to prescribed exercise therapies [56].

### Qualitative explorations of intervention acceptability, adherence, and safety

#### Patient interviews

A purposive sub-sample of participants were identified at the end of the 12-week intervention period for telephone interviews by an independent researcher (AW). The interview was conversational in style and allowed the participant to speak freely about their experience and opinions. Several trigger questions were used to keep the conversation focussed (Additional file 2). The interview was recorded using a digital recorder and data transcribed verbatim by a commercial company (Outsec Services Limited, Swaffham, UK). Thematic analysis was undertaken initially using a thematic network approach [57]. Thematic analyses were independently verified by an additional qualitative researcher. At the third iteration of interpretation, relevant themes were aligned to the Theoretical Framework of Acceptability (TFA) for healthcare interventions [58].

#### Therapist interviews

All therapists were invited to attend a telephone semi-structured interview to explore the acceptability of training and delivery of GREAT Strides. A topic guide informed by the TFA was developed a priori [58] (Additional file 3). One researcher (MS) conducted all interviews, and data were transcribed verbatim by a commercial company (The Typing Works, Middlesex, UK). A deductive thematic analysis was applied in which the TFA was applied as the coding framework. Data were coded into six TFA constructs (affective attitude; burden; intervention coherence; opportunity costs; perceived effectiveness; self-efficacy). Inductive themes within each of the TFA constructs were reviewed amongst the primary coder (MS) and two additional qualitative researchers (LB, EG), until consensus was reached.

#### Fidelity of motivational interviewing delivery

All GREAT Strides sessions were audio recorded. Randomly selected 20-min segments of audio recordings were rated for proficiency of MI delivery by two trained, independent raters using the Motivational Interviewing Treatment Integrity Scale 4.2.1 (MITI, 4.2.1) [59]. Motivational Interviewing technical proficiency (application of MI techniques [range 1–5] 3 = fair proficiency) and relational proficiency (interpersonal style [range 1–5] 3.5 = fair proficiency) were assessed.

### Behaviour change techniques delivery

Bespoke checklists were developed to assess fidelity of delivery of 6 core intervention components delivered in sessions 1, 4 core components delivered in sessions 2–6, 17 BCTs delivered in session 1, and 12 BCTs delivered in sessions 2–6. Two independent assessors trained in applying the Behaviour Change Technique Taxonomy V1 [60] rated the full audio recordings of GREAT sessions. High treatment fidelity was calculated according to whether a minimum of 80% of core components and BCTs within each session were rated as having been delivered by clinicians.

### Candidate primary outcome measures for the future trial

Four measures of lower limb/foot disability with good measurement properties in people with RA [61–65] were selected for evaluation as potentially suitable primary outcome measures for the future randomised controlled trial. The Foot Function Index (FFI) disability subscale is a patient-reported outcome measure designed to measure self-reported foot-related disability. Responses are made using 100 mm visual analogue scales and a summary score obtained by calculating the mean of the nine items, with higher scores indicative of greater foot-related disability [61]. The Patient Reported Outcome Measurement Information System Physical Function Short Form (PROMIS PF-20) is an outcome measure of physical function [62]. Responses are made using twenty 5-level Likert scales, and summary score calculated by summating and converting to a *t* score ranging from 9.2 to 62.7, with lower scores indicative of greater functional impairment. The Recent-Onset Arthritis Disability (ROADles) lower extremity subscale is a measure of lower extremity physical function [63]. Responses are made using four 5-point Likert scales and scores summated and normalised (scores × 0.625) to a summary score ranging from 0 to 10 (higher scores representing poorer status). The 10-m walk test (10MWT) is an objective measure of walking capacity/functional mobility. It is the time taken to walk 10 m with greater time indicative of poorer functional status. Participants’ perceptions of the overall treatment effect were recorded at 12-week follow-up using a 7-point global rating of change scale (defined as change in walking ability [CWA]) as a patient-rated anchor [66] from “very much worse” to “very much better”.

### Recruitment rates, attrition, and data completeness

Future trial feasibility was further explored through examination of recruitment rates, rates of attrition, and rates of completion of outcome measures.

### Study schedule and procedures

Study schedule and procedures details are provided in Table 2.

**Table 2:** Standard Protocol Items: Recommendations for Interventional Trials (SPIRIT) table for study procedures.

	Pre study screening/consent −1	Baseline 0	Compulsory clinical visit 1 T1	Compulsory clinical visit 2 T2	Optional clinical visits 3–6 T3-6	12 weeks follow-up F1
Enrolment						
Eligibility screen	x					
Informed consent	x					
Measurements						
Patient acceptability questionnaire						x
EARS						x
FFI-DS		x				x
PROMIS PF-20		x				x
ROADles		x				x
10MWT		x				x
CWA 7-point scale						x
Qualitative telephone interviews						x
Intervention						
GREAT Strides			x	x	x	

### Statistical analyses

#### Recruitment/retention, demographic, and clinical data

Recruitment, retention, and data completeness rates were calculated using absolute (number of participants [*n*]) and relative (percentages [%]) frequencies. Monthly recruitment rate was estimated using the mean and associated 95% confidence interval (95% CI). Demographic and clinical data are expressed as median (inter-quartile range [IQR]) for data that were not normally distributed, and absolute (*n*) and relative (%) frequencies for nominal data. Intervention acceptability questionnaire item responses and adverse events (AEs) were analysed using absolute (*n*) and relative (%) frequencies. Intervention adherence was analysed using the median (IQR) summary score for the EARS questionnaire.

#### Primary outcome data analyses

In order to select the most appropriate instrument for subsequent project phases, an evaluation of measurement properties including minimal important difference (MID), longitudinal validity and responsiveness to change over 12 weeks was planned, subject to availability and completeness of study data. The MID was estimated by calculating the mean change score in participants who improved according to the CWA (anchor), minus the mean change score in participants who did not improve or whose symptoms worsened, with associated 95% CIs. Linear associations of change scores were determined between candidate outcome measures and the CWA using Pearson’s correlation coefficient and 95% CIs. Responsiveness was to be evaluated using four different effect size statistics: the paired *t* test, Cohen’s *d*, standardised response mean (SRM), and the Guyatt index (GI) [67–70]. The SRM was calculated as the mean change scores between baseline and 12-week follow-up divided by the standard deviation of the differences between the baseline and 12-week follow-up scores. The Guyatt index (GI) represents the magnitude and variability in change scores for an outcome measure relative to the MID of the measure.

### Sample size

An a priori sample size calculation was undertaken based on the evaluation of measurement properties of primary outcome candidates. A sample size of at least *n* = 42 would allow detection of a magnitude of association (correlation coefficient) of at least 0.65 between the selected anchor (CWA) and outcome measures at 5% significance with 80% power, accounting for 20% attrition.

## Results

### Demographics and clinical data

Thirty-five participants were eligible and enrolled. Participant demographics are summarised in Table 3. Participants had a median (IQR) BMI of 26.6 (23.3–31.1), and the majority were in employment (63.6%). Median (IQR) disease duration was 9.1 (4.0–16.2 months), in moderate DAS-28 disease activity states, and most were receiving disease modifying anti-rheumatic drugs (DMARDs) medication (88.6%).

**Table 3:** Baseline demographic and clinical characteristics

Characteristic	Descriptive statistics
Number of participants, *n*	35
Age in years, median (IQR)	60.1 (49.4–68.4)
Female sex, *n* (%)	24 (68.6)
BMI (kg/m^2^), median (IQR)	26.6 (23.3–31.1)
Primary employment status, *n* (%)	
Employed full-time	11 (33.3)
Employed part-time	8 (24.2)
Unemployed	0 (0.0)
Self-employed	2 (6.1)
Retired (because of age)	8 (24.2)
Retired (because of ill health)	2 (6.1)
Student	0 (0.0)
Housewife/husband	1 (3.0)
Other	1 (3.0)
Ethnicity	
British	31 (96.9)
Indian	2 (5.7)
Caribbean	3 (8.6)
Any other white background	1 (3.1)
Disease duration in months, median (IQR)	9.1 (4.0–16.2)
DAS-28 median (IQR)	4.0 (3.1–4.6)
Currently taking DMARDs	31 (88.6)
Currently taking biologic drugs	5 (14.7)
FFI-DS, mean (SD)	34.5 (17.8)
PROMIS-PF-20, mean (SD)	37.6 (9.1)
ROAD-les, mean (SD)	2.5 (2.3)
10MWT in seconds, mean (SD)	11.9 (11.9)

### Intervention feasibility

#### Intervention acceptability

Twenty-three participants completed the intervention acceptability questionnaire at 12-week follow-up (Table 4). The intervention appeared to have excellent acceptability; 21/23 (91.3%) were confident that it could help the problem; 21/23 (91.3%) reported that they would recommend it to a friend; 22/23 (95.7%) indicated it made sense to them.

**Table 4:** Intervention acceptability questionnaire item responses at visit 2 (week 12)

Item	Response	*N* (%) participants
Item 1: How confident are you that treatment can help the problem?	Not at all confident	0 (0.0%)
	Not very confident	1 (4.3%)
	Neither	1 (4.3%)
	Quite confident	11 (47.8%)
	Very confident	10 (43.5%)
Item 2: Would you recommend the treatment to a friend with a similar problem?	Not at all confident	0 (0.0%)
	Not very confident	1 (4.3%)
	Neither	1 (4.3%)
	Quite confident	8 (34.8%)
	Very confident	13 (56.5%)
Item 3: Does the treatment make sense to you?	Not at all logical	0 (0.0%)
	Not very logical	0 (0.0%)
	No opinion	1 (4.3%)
	Quite logical	10 (43.5%)
	Very logical	12 (52.2%)

### Intervention safety

One participant reported mild transient post-exercise soreness which was an expected event of interest. One SAE was reported that was unrelated to the intervention or study participation.

### Intervention adherence

**Fig. 2:**
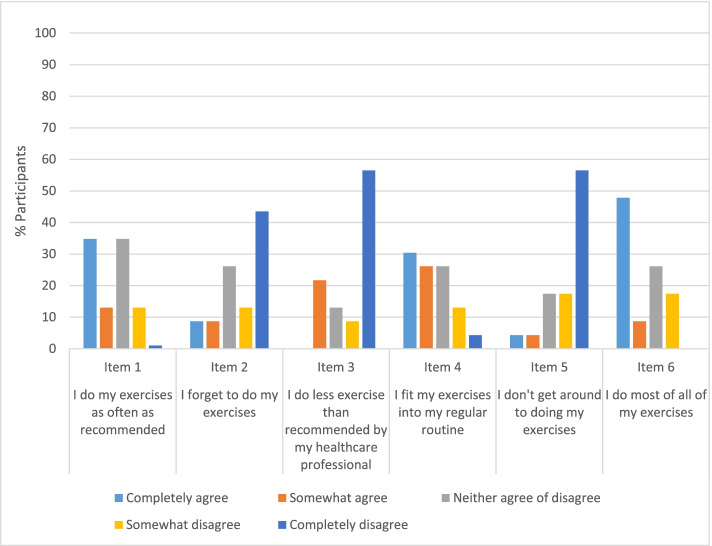
Exercise Adherence Rating Scale (EARS) item responses at visit 2 (week 12)

Twenty-three participants completed the EARS at 12-week follow-up (Fig. 2), and a median (IQR) score of 17.5 (12.5–22.5) indicated good overall adherence to the intervention.

### Participant interview findings

All but 2 of the 12 interview participants stated in the interview that they had continued with the intervention after the 12-week study period. Three global themes emerged from the data; intention and motivation, satisfaction of experience, and barriers to continuation which were aligned to relevant TFA constructs (Table 5).

**Table 5:** Themes, TFA constructs, and supportive narratives from participant interviews

Themes	TFA constructs	Supporting narrative
Intention and motivation	Affective attitude	“*I wanted to see what my limits were and so it was a no brainer to give it a go*” 010
		*“ … it was at diagnosis that my whole world started to change … it created an opportunity to gain some control back” 009*
		“*I am self-employed, so I've got to work, I've got to do it. So, it's in my interests to get myself as well as I can be*” 003
		“*it was a challenge, but as I got on, I got faster and better at it and I was quite happy with that*.” 004
		“*Sometimes it’s very easy to just sit. And having to do those, sort of got me going, got me moving*.” 001
Satisfaction of experience	Perceived effectiveness	“*One of the tests where you had to put one foot in front of the other … I struggled with that as far as balance was concerned initially and the programme sort of improved that*” 001
		“ *… Once this [RA] came on, I couldn't do it [walking with friends]. Now, since I've gone on the gait project, I've started again, and I've done about three- or four-mile walks*,” 008
		“ … *I feel my heart beating so it’s got to be good for general fitness … I enjoy it and it lifts my mood*” 010
		*“It doesn’t work miracles overnight … it was a month and two weeks, that I could really see the difference*” 004
		“*Obviously, I'm a bit stiff and it sort of limbers up a little bit, gets you going a little bit. I suppose it was just to get me moving*” 006
Barriers to continuation	Burden	“*There's not space in the house, it's too small. I've only got my living room and my kitchen, and then the living room is only small, and it's got furniture and everything in it, so there's not … and it's not safe to do anything, to be honest*” 003
		“*Sometimes I’d do consecutive days and then miss a couple of days and other times I stuck to the three a week.*” 001
	Opportunity costs	“*Moving to a new house is an obstacle … But maybe once I've moved house and I've got less house work and gardening, I'm going to really go for it and I'm going to try and do it as often as possible* … .” 009
		*“ … if I did do it at home I would find it difficult with the kids … its easier for me to go to a gym (after the hospital sessions) …* ” 010

#### Intention and motivation

There appeared to be a positive attitude as a character trait observed in all interview participants to try the intervention (affective attitude). This positive attitude emerged from determination and could have enabled adherence to the intervention. Most participants were seeking a solution and/or a challenge. For some, there were motivations towards being well enough to work as a result of doing the exercises.

#### Satisfaction of experience

Positive experiences of undertaking the exercises were described and perceived benefits appeared to maintain motivation (perceived effectiveness). Progression was apparent for the majority and a sense of achievement was described. There were perceptions of benefits amongst several participants such as improved movement ability, flexibility, balance, muscle strength, and resumption of social activity. Some participants highlighted negative experiences such as recurrence of previous injuries.

#### Barriers to continuation

Having enough space to carry out the intervention was one of the major issues identified (burden). However, participants acknowledged that the flexibility of the intervention was useful. Major life events such as moving to a new house and everyday responsibilities such as household chores were identified as main time sacrifices required to do the exercises (opportunity costs).

### Therapist interview findings

Nine therapists (four physiotherapists, five podiatrists) participated in semi-structured interviews. Key barriers and enablers with regards to the acceptability of the training and intervention were identified (Table 6). Therapists liked the supportive training environment (affective attitude) and reported role play exercises aided confidence in applying MI and BCTs (self-efficacy). The lack of time available to attend training was considered problematic (opportunity costs). All therapists valued the opportunity to provide individualised care (intervention coherence). Barriers associated with acceptability included the use of trial-related materials (e.g. checklist) during intervention delivery (burden) and time delay between receiving training and intervention delivery (perceived effectiveness).

**Table 6:** Themes, TFA constructs, and supportive narratives from therapist interviews

	TFA constructs	Supporting excerpts
Training acceptability barriers	Opportunity costs	*“In a busy NHS clinic, there's always pressure on patients and waiting times, two days out of a clinic is a big ask it did have a knock-on effect, and a potential impact on patient care”* (01_002)
Training acceptability enablers	Affective attitude	*“it was a really nice small, friendly, informal environment”* (03_003)
	Perceived effectiveness	*“The packaging that we got was excellent, there wasn't anything that was left out, I actually refer to training materials a lot in clinic, everything was really well done”* (03_004)
	Self-efficacy	*“I felt confident doing the MI practice because I’ve had training before”* (03_003)
Intervention delivery acceptability barriers	Burden	*“It definitely felt like there’s a lot of paperwork. Putting it all together in a sequence with all the paperwork in front of you … that felt like there was quite a lot to do for one appointment”* (03_003)
Intervention delivery acceptability enablers	Intervention coherence	“*The follow-up sessions were good to check if we’d changed something in the second consultation and I just wanted to make sure that they were managing the alterations that we’d agreed and to make sure that they were happy in what they were doing*” (03_001)
	Perceived effectiveness	*“The ones that were motivated and committed to it and did it, were thrilled at how quickly they started to pick up the exercises. They could see the changes in themselves, so, it definitely is effective” (02_001)*

### Intervention fidelity

Four physiotherapists and two podiatrists delivered 78 GREAT Strides sessions across three centres in the UK (see Additional file 4). Audio recordings of the GREAT Strides intervention for 28 participants across 64 sessions were considered for the assessment of fidelity. A sample of 37 (50%) of sessions 1–6 delivered across the three sites were coded for MITI delivery. Good inter-rater reliability was achieved, 73% (CI 0.68–0.78). Relational (mean [SD] 4.38 [0.844]) and technical (mean [SD] 4.19 [0.837]) aspects of MI were delivered with proficiency. Data from 28 participants across 55 sessions were rated for core components and BCTs. The 6 core components and 7 BCTs in session 1 were delivered with high (≥ 80%) treatment fidelity but 10 further BCTs were not consistently delivered. In session 2, 4 core components and 4 BCTs were delivered with high fidelity, but another 8 BCTs were not consistently delivered. In session 3, three core components and two BCTs were delivered consistently. In session 4, 3 core components were delivered with high fidelity, but none of the 12 BCTs were delivered consistently. For sessions 5 and 6, only 2 core components were delivered consistently, with 6 out of 12 BCTs for session 5 being delivered with high fidelity, and 2 BCTs out of 12 being delivered with high fidelity for session 6

### Evaluation of future randomised trial feasibility

#### Primary outcome measure candidates’ evaluation

**Fig. 3:**
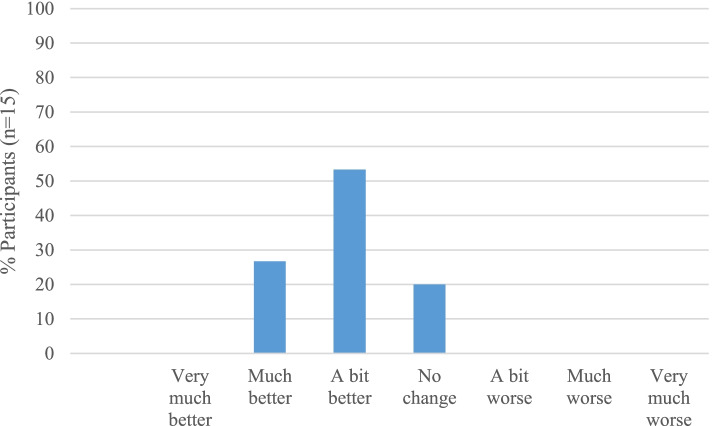
CWA (7-point Likert scale) at 12 weeks

Mean change scores and associated effect sizes for estimating responsiveness are presented in Table 7 for the 15 participants who had complete data. Participants’ perceptions of change in walking ability at 12 weeks are presented in Fig. 3. Change scores for outcome measures were all in the expected direction (improvement). Effect sizes were within the small to medium range. The PROMIS-PF-20 and ROAD-les appeared to be modestly more responsive relative to the FFI-DS and 10MWT. It was not possible to calculate the Guyatt Index due to the inability to calculate MID values for the PROMs as a result of the distribution of the corresponding CWA data.

**Table 7:** Change scores summarised for primary outcome measure candidates

	Mean change 12 weeks	*T*-statistic (*p* value)	Cohen’s *d* (95% CI)	SRM
FFI-DS	– 4.14	– 1.07, *p* = 0.296	0.24 (– 0.22, 0.69)	– 0.22
PROMIS-PF-20	1.85	2.59, *p* = 0.017	– 0.28 (– 0.50, – 0.06)	0.54
ROAD-les	– 0.79	– 2.36, *p* = 0.028	0.44 (0.05, 0.84)	– 0.49
10MWT in seconds	– 2.75	– 1.03, *p* = 0.316	0.23 (– 0.23, 0.70)	– 0.21

The mean change from baseline summarised for the candidate primary outcome measures for those with complete outcome data at baseline and 12-week visits. Responsiveness statistics presented include the paired *t* test statistic (and p-value), Cohen's D (95% CI) and the standardised response mean (SRM)

Missing data for the CWA score and observed distributions across ordered categories of the Likert scale limited our ability to perform a priori planned analyses. For the 15 participants who completed all outcome measures at 12 weeks, results suggest that the FFI-DS, PROMIS PF-20 and 10MWT measures performed best in terms of theoretical consistency, direction of change and longitudinal validity (Tables 7 and 8). The ROADles lacked theoretical consistency as a greater improvement was observed in those classed as “not improved” relative to “improved” according to the CWA (Table 8 and Additional file 5).

**Table 8:** Mean change according to CWA improvement for primary outcome measure candidates

Outcome measure	All (*n* = 15)	Improved (*n* = 12)	Not improved (*n* = 3)	Mean difference improvers versus non-improvers (95% CI)	*r*
FFI-DS	– 2.27	– 2.63	– 0.8	– 1.83 (– 32.58, 28.92)	– 0.12
PROMIS PF-20	1.69	1.8	1.23	0.57 (– 6.24, 7.37)	0.16
ROADles	– 0.75	– 0.73	– 0.83	0.10 (– 2.35, 2.56)	0.17
10MWT	– 0.91	– 1.03	– 0.43	– 0.60 (– 2.42, 1.22)	– 0.28

#### Recruitment, retention, and data completeness

**Fig. 4:**
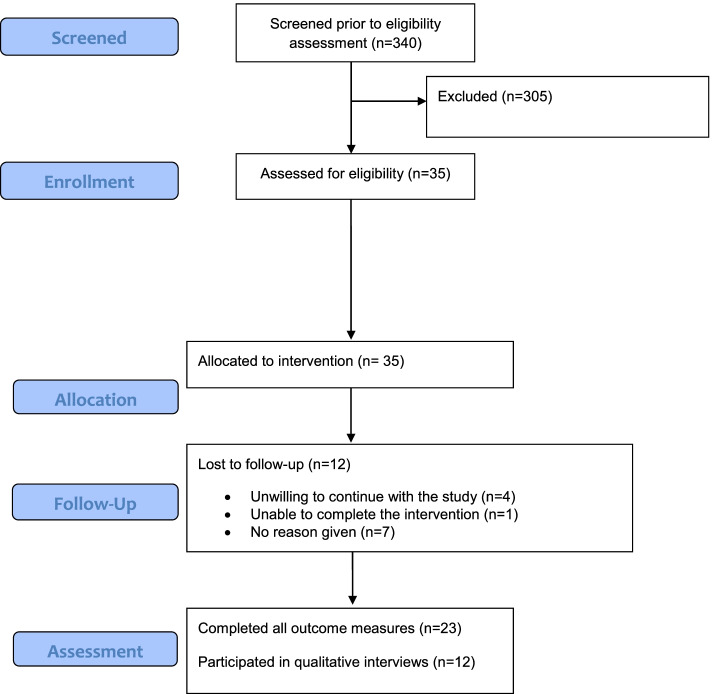
Study flowchart for recruitment

Recruitment ran from June 2018 to March 2019, and follow-ups were completed July 2019. A total of 340 patients were screened and 35 participants were identified as eligible and enrolled (Fig. 4). Monthly participant recruitment rate was 3.5 (95% CI 2.46, 4.79). Twelve participants withdrew from the study, of which 6 were lost to follow-up. The mean (SD) time from baseline to study withdrawal was 80 (49.9) days. Four participants stated they were unwilling to continue in the study, and one was unable to complete the intervention. A total of 23 (65.7%) participants completed follow up at 12 weeks from baseline. All 23 participants completed all outcome measures (*n* = 23, 100%) with the exception of the CWA scale, which was completed by 15 participants (*n* = 8, 34% missing).

## Discussion

The results of this study found that the GREAT Strides programme was viewed as acceptable by patients and therapists, and was delivered with good MI proficiency and high core components fidelity, good patient adherence, and no safety concerns. Some adaptations to the intervention and therapist training are required to optimise delivery of BCTs.

The feasibility of a future randomised trial included identification and justification of a suitable primary outcome measure, somewhat acceptable recruitment rates and data completeness were demonstrated but refinements are required.

### Intervention acceptability

The acceptability of the intervention amongst participants was high. This was largely corroborated by our interview findings. These positive findings appear to be driven initially by a positive affective attitude character trait and a willingness to try to improve and regain control, and subsequently experiences of perceived effectiveness of the intervention which was also reflected by therapists. These findings were encouraging given the reported variable success of interventions involving behaviour change for promoting exercise and physical activity [71]. References to perceived improvements in key therapeutic targets that the intervention was designed to address such as improved mobility, balance, and strength provide further intervention credibility. Whilst there was no explicit mention of the behaviour change techniques that were embedded within the delivery of GREAT Strides, qualitative explorations suggest that participants may have experienced improved self-efficacy and ability to overcome barriers to completion. Indeed, interventions which enable barriers to exercise and physical activity to be overcome have been identified as essential to help sustain behaviour change [23]. However, some barriers to completion were identified.

Intervention training was largely viewed as a positive experience by therapists. Previous experience of MI and a desire integrate psychological approaches into practice appeared to result in greater confidence in intervention delivery. The training content and delivery was described as comprehensive but the requirement to attend two face-to-face sessions and time pressures were identified. In isolated cases, there was a gap between completion of training and first delivery of the intervention, which needs to be addressed where possible. Future therapist training has been refined to be delivered online and, includes self-directed learning, synchronous online practice sessions with support, and refinement of therapist support materials.

Intervention delivery was considered to be coherent and perceived as effective amongst therapists. However, some challenges were identified concerning the need for follow-up appointments within a 2-week timeframe for therapists with busy caseloads, and the burden of paperwork required.

### Safety and adherence

The intervention was well tolerated by participants, with only one report of an SAE unrelated to the intervention, and one report of an expected adverse event (AE) of post-intervention soreness. These findings were largely corroborated by the qualitative exploration where reference is made to some post-intervention ankle soreness (one participant with a previous history of an ankle injury), and additional reference to possible safety concerns where there was a lack of sufficient floor-space at home. These findings agree with recent evidence suggesting walking exercise programmes for people with early and established RA appear to be well tolerated [72, 73].

### Adherence

Good adherence to the intervention was demonstrated, with a median EARS score of 17 from a possible 24. Interpretation of this score is difficult due to a lack of comparable UK data from the early RA population, however recent research from Brazil and Nepal suggest cut-off scores of 17 and 17.5 respectively to discriminate adherent and non-adherence participants with respect to prescribed exercise [74, 75].

### Intervention fidelity

The fidelity assessment methods proved to be appropriate, successful and therefore suitable for use in a future trial. Clinicians were able to deliver the most components of GREAT-Strides (MI, all core components, and some BCTs) with high fidelity. However, not all BCTs were delivered with high fidelity. The intervention will be simplified to specify mandatory and optional BCTs, depending on needs of individual participants, as previous research shows interventions with seven or fewer BCTs may be more effective at enhancing adherence [76]. Fidelity results and clinician interviews have enabled training refinement, including demonstration videos to support therapists deliver the intervention. Results also suggest that tailoring the training accounting for prior experience in delivering MI may be required.

### Primary outcome measures

Results of the analyses of the primary outcome candidates were considered by the Trial Management Group in order to recommend which measure would be taken forward to a future main trial. Similarities were observed across the 3 of the 4 primary outcome measure candidates in terms of their responsiveness and theoretical consistency in terms of anticipated direction and magnitude of change over time. The ROADles was not recommended for the future main trial due to its lack of theoretical consistency. Given the similarities observed for the 3 remaining measures, practicalities and relative simplicity of measures were considered as recommended by the COnsensus-based Standards for the selection of health Measurement INstruments (COSMIN) [77]. The 10MWT was identified as having additional practical implications, requiring face to face assessment with sufficient floor space. The PROMIS-PF-20 performed well overall in terms of measurement properties, but issues were identified with the additional steps required for scoring (conversion to *t* scores) prior to analysis and concerns raised regarding interpretability. Therefore, the FFI-DS was preferred due to its largely desirable measurement properties, ease of completion and scoring, widespread use, and clinical relevance. A limitation of the primary outcome analysis was that, due to unexpected data distribution where too few participants scored in the “slight improvement” category of the global rating scale, it was not possible to calculate MID robustly. This meant that it was not possible to calculate and present GI values as planned.

### Recruitment and retention

Despite high prevalence of foot disease and impaired lower limb function in early RA, our recruitment rate for the GREAT Strides programme was lower than anticipated, with an average of 3.5 participants per month. Poor enrolment and attrition rates have been reported in other feasibility studies involving behaviour change in this population [78]. Through evaluation of screening logs and dialogue with site personnel, we established one of the limiting factors was confirmation of meeting ACR 2010 classification criteria for RA, which is often not recorded in routine care and the inclusion criterion for the future main trial will be amended so that patients with a clinician diagnosis of RA can be included.

The prevalence of disease-related foot and ankle involvement and thus the pool of potentially eligible participants appeared to be far lower than anticipated. As a result, we anticipate that more recruitment sites will be required for the future main trial to achieve desired recruitment numbers. Moreover, the mailshot approach to recruitment appears to be an essential component of the recruitment strategy to supplement recruitment from the rheumatology outpatients setting. Recruitment initially took place in “early arthritis” clinics only, where patients in the first year following their RA diagnosis were being seen by clinicians. Such patients are coming to terms with a life changing diagnosis and experience a high volume of clinical appointments to control inflammatory disease activity, and therefore may be less likely to agree to participate in research. Future recruitment will be extended to established RA clinics in order to identify additional potentially eligible participants within 2 years post-diagnosis.

Attrition rate was higher than anticipated at 34.3%. The reasons for this remain largely unclear, as the majority of participant attritions were lost to follow-up without giving a reason (*n* = 6). This feasibility study necessitated in-person follow-ups for collection of the performance-based outcome measure 10MWT at 12 weeks. However, with refinement of outcome measures, these could be collected remotely which may improve retention and data completeness. The addition of short message service reminders could also improve follow-up adherence [79]. In addition, based on the number of GREAT intervention sessions delivered in the feasibility phase, where only one patient received sessions 5 and 6, reducing the intervention to two compulsory plus further two optional sessions seems sufficient.

## Conclusions

GREAT Strides was viewed as acceptable by patients and therapists, was mostly delivered as intended, with good patient adherence and no safety concerns. Recommendations are provided for primary outcome selection for a future trial. Recruitment and attrition rates may improve with refinement of the intervention, follow-up procedures, and eligibility criteria, and should be further evaluated via a pilot trial.

## Supplementary Information

**Additional file 1.** Events of special interest. Table of events of special interest for the GREAT Strides intervention.

**Additional file 2.** Interview Script Participants. This document is the interview script/guide used by the facilitator to interview participants.

**Additional file 3.** Interview Script Therapists. This document is the interview script/guide used by the facilitator to interview therapists.

**Additional file 4.** Intervention fidelity results and reliability. Table of inter-rater reliability, and fidelity of core components and Behaviour Change Techniques delivered across all GREAT Intervention sessions.

**Additional file 5.** PROM scores by CWA. Table of mean change at 12-weeks summarised for the candidate primary outcome measures, split by response to the CWA 7-pt Likert scale. Estimated differences (and 95% CI) summarised for the mean change at 12-weeks per 1-unit increase in the CWA. E.g. The estimated difference between those who responded 'No change' versus 'A bit better' or 'A bit better' versus 'Much better'.

**Additional file 6.**

## Data Availability

The data that support the findings of this study are available from the corresponding author pending reasonable request

## Abbreviations

10MWT: 10-metre walk test
ACR: American College of Rheumatology
AE: Adverse event
BCT: Behaviour change technique
BMI: Body mass index
CI: Confidence interval
COSMIN: COnsensus-based Standards for the selection of health Measurement INstruments
CWA: Change in walking ability
DAS-28: Disease activity score 28
DMARD: Disease modifying anti-rheumatic drugs
DVD: Digital versatile disc
EARS: Exercise adherence rating scale
FFI-DS: Foot function index disability subscale
GI: Guyatt index
GREAT: Gait rehabilitation in early arthritis trial
IQR: Inter-quartile range
MI: Motivational interviewing
MID: Minimal important difference
MITI: Motivational interviewing treatment integrity scale
MRC: Medical Research Council
NHS: National Health Service
PPI: Patient and public involvement
PROMIS PF-20: Patient Reported Outcome Measurement Information System Physical Function Short Form
RA: Rheumatoid arthritis
ROADles: Recent-Onset Arthritis Disability lower extremity subscale
RPE: Rating of perceived exertion
SAE: Serious adverse events
SD: Standard deviation
SPIRIT: Standard Protocol Items: Recommendations for Interventional Trials
SRM: Standardised response mean
TBP: Theory of planned behaviour
TFA: Theoretical framework of acceptability
