# Supplementary material for: Gait rehabilitation for foot and ankle impairments in early rheumatoid arthritis: a feasibility study of a new gait rehabilitation programme (GREAT Strides)
Source: Pilot Feasibility Stud. 2022 May 30;8:115. doi: 10.1186/s40814-022-01061-9 (PMC9150324; doi:10.1186/s40814-022-01061-9)
Supplement: Supplementary file 1 — Additional file 1. Events of special interest. Table of events of special interest for the GREAT Strides intervention. [file 40814_2022_1061_MOESM1_ESM.docx]

Additional File 1. Events of special interest

| Events of special interest |
| --- |
| Transient post exercise soreness  Post exercise stiffness  Post exercise fatigue  Post exercise trips, slips and/or falls  Temporary exacerbation of disease-related inflammatory pain during exercises  Trips, slips and/or falls during setup of circuit, during exercises, and/or clearing away the circuit setup  Temporary musculoskeletal pain from setup of circuit at home  Perceptions of new instance of disease flare resulting from undertaking gait rehabilitation circuit |
