# Supplementary material for: Gait rehabilitation for foot and ankle impairments in early rheumatoid arthritis: a feasibility study of a new gait rehabilitation programme (GREAT Strides)
Source: Pilot Feasibility Stud. 2022 May 30;8:115. doi: 10.1186/s40814-022-01061-9 (PMC9150324; doi:10.1186/s40814-022-01061-9)
Supplement: Supplementary file 2 — Additional file 2. Interview Script Participants. This document is the interview script/guide used by the facilitator to interview participants. [file 40814_2022_1061_MOESM2_ESM.docx]

Interview Guide

Title of study - **An exploration of the acceptability of the new gait rehabilitation intervention by identifying participants’ (patients) views and experiences of receiving the intervention.**

**Researcher prompt**:

Check that you have a signed copy of the participant consent form

Introduce yourself; orientate to topic/reason for interview; how long you think you will be; reassurance re: anonymity/confidentiality and can stop at any time without giving reason. Check they have understood the PIS and if there are any questions or points that need clarification. Confirm consent before proceeding. Reassure the participant that there are no right or wrong answers and the researcher simply wants to hear their opinions and views. Remind the participant of their right to withdraw from the study at any time, without reason. Intermittently check that the participant is OK to continue with questioning with breaks offered if need be.

Opening questions

1. What were your personal experiences of walking *before* the gait training?
2. What are your perspectives and opinions of the gait training you have been doing?
3. Have there been any immediate benefits to you?
4. What are the potential restrictions for embedding this into your daily routine?

Further trigger questions will be used in relation to each of these areas -

Do you have any past problems with walking? (1)

Do you have any current problems with walking? (1)

What are problems (if any) with walking that you think are most problematic in your daily life. (1)

Did you understand the purpose of gait training? [if no – what would have helped? If yes what was the most important information] (2)

Did the information provided to you motivate you to complete the training? (2) [If no – why? If yes – what specifically?]

Are there any aspects of the gait training that you find difficult? [if yes – ask specifically ; If no – what were the easiest aspects] (2)

Were there any aspects of this training which made you feel unsafe? (2)

Do you think these immediate benefits will last? (3)

What do you think you need to do in order to maintain these benefits? (3)

Did you have any issues with the gait training?(3)

What might you want in relation to gait training to help with fitting this into your day to day routines? (4)

Is there anything that stops you completing the training? (4)

In order to keep the conversation going, open ended questions will be used following statements made by the participants to encourage more indepth understanding by the researcher. Examples of these are:

How did you feel about that?

Did that have any other meaning to you?

How important is that?

What has been your experience to date?

What would you like to be able to do?

What other factors would help?

Do you have anything else to comment on?

Prompt for researcher

Thank the participant for their time. Reinforce re anonymity and what will happen with the data collected.

Title of Study - **An Exploration of the acceptability of the new gait rehabilitation intervention by exploring clinicians (Physiotherapists and podiatrists) views and experiences of delivering the intervention**

**Researcher prompt**:

Check that you have a signed copy of the participant consent form

Introduce yourself; orientate to topic/reason for interview; how long you think you will be; reassurance re: anonymity/confidentiality and can stop at any time without giving reason. Check they have understood the PIS and if there are any questions or points that need clarification. Confirm consent before proceeding. Reassure the participant that there are no right or wrong answers and the researcher simply wants to hear their opinions and views. Remind the participant of their right to withdraw from the study at any time, without reason. Intermittently check that the participant is OK to continue with questioning with breaks offered if need be.

**Opening question-**

What was your experience of delivering the gait rehabilitation intervention?

**Additional questions**

What is your opinion of patient acceptability of this intervention?

What do you consider the most important factors which need to be considered in respect of adherence?

From your perspective what are the main (potential) benefits of this intervention?

Were there any aspects of it where there were (or might have been) issues with safety?

What do you think the main obstacles to patient adherence may be?

Did you have to make any modifications to the intervention? If so what were they and why dod you have to make these modifications?

Are any refinements to the intervention needed?

Do you have any other comments?
