# Supplementary material for: Gait rehabilitation for foot and ankle impairments in early rheumatoid arthritis: a feasibility study of a new gait rehabilitation programme (GREAT Strides)
Source: Pilot Feasibility Stud. 2022 May 30;8:115. doi: 10.1186/s40814-022-01061-9 (PMC9150324; doi:10.1186/s40814-022-01061-9)
Supplement: Supplementary file 3 — Additional file 3. Interview Script Therapists. This document is the interview script/guide used by the facilitator to interview therapists. [file 40814_2022_1061_MOESM3_ESM.docx]

**Clinicians experiences and acceptability of receiving the GREAT training and delivering the gait rehabilitation intervention**

**Topic guide**

Thank you for taking the time to participate in this telephone interview.

Just to double check before I continue, are you still happy for me to record this interview?

Great, so the interview will last between 40-60 minutes. Before starting, I’ll give you a summary of the structure of the interview. This interview is structured into three different parts. In the first part we will focus on your experience and thoughts of the GREAT ***training*** you received. In part two we will discuss your experience of ***delivering*** the gait rehabilitation and in part three we will ask your thoughts about the ***trial processes*** involved with delivering the GREAT intervention.

**Background questions**

1. We’ll begin the interview by asking you some questions about your role. Can I firstly ask how long you have been qualified and practising as a physio/podiatrist?

- *(clarify if therapist is a physio or podiatrist)*

1. How long have you been working with people with MSK conditions/ RA?
2. Have you had any experience of supporting patients in changing their own behaviour?

- *If so can you tell me more?*

1. Prior to the GREAT intervention, have you had any training/ experience of delivering motivational interviewing?

- *Can you tell me more?*

1. Throughout the GREAT trial, how many patients have you seen so far?

- *On average do you remember how many sessions you delivered per patient?*
- *Following on from that, with the optional sessions – did you deliver most of these face to face, over the telephone or was it a mixture?*

1. Okay, so before asking questions relating to the training you received, can I just ask when you completed the two-day training sessions?
2. and how soon after did you first deliver the GREAT intervention?
3. What are your thoughts on the time between receiving the training and delivering the intervention?

- Do you think this had an impact on how you delivered the GREAT intervention?

**Part 1: Acceptability of GREAT training**

1. How acceptable did you find the GREAT training?

- *We are interested in what acceptability might mean to you, so any initial thoughts you might have?*

1. Can you talk me through your overall experience of the GREAT intervention training and support materials you received?

- *What are your thoughts on the two-day training session?*
- *Do you feel it was appropriate to take two days out of your clinical practice to attend the sessions?*
- *How much effort did it require to attend the sessions?*
- *what are your thoughts on the clinician manual that was provided?*
- *What are your thoughts on the patient support materials? (i.e. patient DVD and patient manual)*
- *Can you describe to me what you feel was the purpose of you attending the training?*
  - *How do you think attending the training sessions may help you deliver the GREAT intervention?*

1. We are interested in your thoughts about the training days that you attended. Was there anything that you particularly liked or disliked about the training?

- *Are there any parts that stand out?*

1. How confident did you feel in practising some of the MIT and BCT techniques in the training days?

- *Can you tell me more? What was Easy/ Difficult?*
- *For example, MIT techniques and/or BCT techniques*

1. In between the two training sessions did you do any of the homework that was suggested? (i.e. viewing the MIT videos?)

- *Was this more effort than you anticipated?*
- *Can you think of any ways that the training you received could be changed or improved?*

1. To what extent do you think the GREAT therapist training prepared you to deliver the GREAT intervention?

- *Can you tell me more?*
- *Why do you say that?*

1. Did attending the training sessions interfere with your other workload priorities?

- *If so, can you tell me more?*

1. Having completed the training, has the training alerted your practice at all?

- *How would you say some of techniques fit with your general approach to patient consultations?*
- *Can you tell me more?*
- *Do you feel that there are any ethical issues that need to be considered?*

**Part 2: Acceptability of delivering gait rehabilitation intervention**

So now we will move onto part two of the interview. Some of these questions are very similar but we are interested in your thoughts on your experience and acceptability of delivering the gait rehabilitation intervention.

1. To what extent did you find delivering the gait rehabilitation intervention acceptable?
2. Can you talk me through your overall experience of delivering the gait rehabilitation intervention?
3. How did you find delivering the initial two compulsory sessions?

- *And how did you find delivering the additional sessions?*

1. Was there anything in particularly that you liked or disliked about delivering the gait rehabilitation intervention?

- *Are there any parts that stand out?*

*Follow on with prompts if participant mentions specifics e.g. using the checklist*

- *With regards to the additional sessions, sessions 3-6 in your opinion did you find patients needed these?*
  - *Can you tell me more?*
- *With regards to the checklist that was provided, how useful was it in the sessions as a prompt?*
  - *Can you tell more?*
- *Do you think there was anything missing from the checklist that may have helped in delivering the intervention?*

1. In your opinion how easy or difficult was it to deliver the gait rehabilitation intervention?

*What was easy/difficult?*

- *How much effort was required to deliver the gait rehabilitation intervention?*
- *Can you tell me more?*

1. Can you briefly tell me how you think delivering the gait rehabilitation intervention will help patients manage their symptoms?

- *Can you tell me more?*

1. To what extent do you feel delivering the gait rehabilitation intervention has any ethical implications for patient care?

- *Can you tell me more?*

1. How do you think patients have responded to the intervention?

- *To what extent do you think delivering the gait rehabilitation intervention has the potential to make a difference to patients’ symptoms?*
- *To what extent do you think patients are likely to engage with the gait exercises?*

1. Did delivering the gait rehabilitation intervention interfere with your other work load priorities?

- *If so, can you give me an example?*

1. How confident did you feel about delivering the gait rehabilitation intervention?

- *Can you tell me more?*
- *Did your confidence change as you saw more patients?*

1. Did you adapt any parts of the gait rehabilitation intervention?

- *If yes, can you give me an example (e.g. why and what)*
- *How much time did this take?*
- *Would you normally spend time adapting an intervention/ patient consultation?*

1. What type of on-going support do you think will help with the delivery of the GREAT intervention?

- *Can you tell me more?*
- *What are your thoughts on online support materials i.e. videos?*
- *Refresher training?*
- *If there was on-going support available, do you think you would have accessed it? How would this fit into your workload?*
- *Which methods of on-going support would you prefer?*

1. In your opinion, what influenced patients adhering to the intervention?

- *Can you give me an example?*
- *How do you think patient adherence could be improved?*

**Part 3. Trial processes**

We’re coming onto the final section of the interview. Here we are interested in hearing about your thoughts on the trial processes associated with the GREAT intervention, specifically if there are any ways in which the processes could be improved.

1. Could you briefly describe the trial processes for your site once the patient had been screened?

- *How was the patients interest/ consent communicated to you?*
- *Do you think this could be improved?*

1. How easy or difficult did you find it to complete any paperwork associated with the trial?

- *Can you give me an example?*
- *Do you think this could be improved?*

1. How did you find the process of collection and transferring of audio data?

- *Did your site have a system in place?*
- *Can you tell me more?*

1. Do you have any additional comments regarding the GREAT training, delivering the intervention and trial processes?

Thank – you that brings us to the end of the interview.
