# Supplementary material for: Gait rehabilitation for foot and ankle impairments in early rheumatoid arthritis: a feasibility study of a new gait rehabilitation programme (GREAT Strides)
Source: Pilot Feasibility Stud. 2022 May 30;8:115. doi: 10.1186/s40814-022-01061-9 (PMC9150324; doi:10.1186/s40814-022-01061-9)
Supplement: Supplementary file 4 — Additional file 4. Intervention fidelity results and reliability. Table of inter-rater reliability, and fidelity of core components and Behaviour Change Techniques delivered across all GREAT Intervention sessions. [file 40814_2022_1061_MOESM4_ESM.docx]

Additional file 4. Inter-rater reliability, and fidelity of core components and Behaviour Change Techniques delivered across all GREAT Intervention sessions

| GREAT Session | Sessions rated | Inter rater agreement | Percentage agreement | Weighted Kappa Coefficient | % of BCTs delivered with ≥80% fidelity | BCTs delivered with ≥80% fidelity | % of core components delivered with ≥80% fidelity | Core components with >80% fidelity |
| --- | --- | --- | --- | --- | --- | --- | --- | --- |
| **Session 1**  *6 core components*  *17 BCTs* | 22/ 28 | 432/506 | 85 % | K_w_ = 0.67, p < .001,  95% CI = [.60-.74] | 32%  7/17 | Information about health consequences  Demonstration of the behaviour  Instruction on how to perform behaviour  Behavioural practice/rehearsal  Feedback on behaviour  Goal Setting (behaviour)  Action planning | 100%  6/6 | Gives a short overview of the GREAT intervention  Conducts a brief clinical assessment  Conducts Psychological assessment using MI  Completes worksheets from support booklet  Gives patient DVD and manual  Confirms appointment for session 2 |
| **Session 2**  *4 core components*  *12 BCTs* | 14/15 | 194/224 | 87% | K_w_ = 0.73 p < .001,  95% CI = [.64 -.82] | 28%  4/12 | Review behavioural goal  Feedback on behaviour  Behavioural practice/rehearsal  Action planning | 75%  3/4 | Reviews progress on gait circuit  Checks gait circuit progression  Completes/ reviews worksheets from patient support booklet  Plans for self-management |
| **Session 3**  *4 core components*  *12 BCTs* | 10/11 | 136/160 | 86% | K_w_ = 0.79p < .001,  95% CI = [.71-.88] | 20%  2/12 | Verbal persuasion about capability  Review behavioural goal | 75%  3/4 | Reviews progress on gait circuit  Plans for self-management |
| **Session 4**  *4 core components*  *12 BCTs* | 7/8 | 98/112 | 88% | K_w_ = 0.93., p <.001  95% CI = [.83-.1.02] | 0/12 |  |  | Reviews progress of gait circuit  Completes/ and or reviews the worksheet from the patient support booklet  Signposts to local walking groups |
| **Session 5**  *4 core components*  *12 BCTs* | 1/1 | 9/16 | 56% | K_w_ = 0.75., p <0.05  95% CI = [.36 - 1.14] | 50%  6/12 | Verbal persuasion about capability  Reviews behavioural goal  Problem solving  Social support (unspecified)  Goal setting (behaviour)  Action planning | 50%  2/4 | Reviews progress on gait circuit  Checks gait circuit progression |
| **Session 6**  *5 core components*  *12 BCTs* | 1/1 | 11/17 | 65% | K_w_ = 0.78., p <0.05  95% CI = [.46-1.12) | 20%  2/12 | Verbal persuasion about capability  Reviews behavioural goal | 50&  2/4 | Reviews progress of gait circuit  Checks gait circuit progression |
