# Supplementary material for: Gait rehabilitation for foot and ankle impairments in early rheumatoid arthritis: a feasibility study of a new gait rehabilitation programme (GREAT Strides)
Source: Pilot Feasibility Stud. 2022 May 30;8:115. doi: 10.1186/s40814-022-01061-9 (PMC9150324; doi:10.1186/s40814-022-01061-9)
Supplement: Supplementary file 5 — Additional file 5. PROM scores by CWA. Table of mean change at 12-weeks summarised for the candidate primary outcome measures, split by response to the CWA 7-pt Likert scale. Estimated differences (and 95% CI) summarised for the mean change at 12-weeks per 1-unit increase in the CWA. E.g. The estimated difference between those who responded 'No change' versus 'A bit better' or 'A bit better' versus 'Much better'. [file 40814_2022_1061_MOESM5_ESM.docx]

Additional File 5

| Outcome measure | No change (n=3) | A bit better (n=8) | Much better (n=4) | Estimated difference in mean  change at 12-weeks per  1-unit increase in CWA  (95% CI) |
| --- | --- | --- | --- | --- |
| FFI-DS | -0.8 | -1.08 | -5.75 | -2.64 (-33.2, 27.91) |
| PROMIS PF-20 | 1.23 | 1.35 | 2.7 | 0.78 (-5.95, 7.51) |
| ROADles | -0.83 | -0.94 | -0.31 | 0.29 (-2.14, 2.71) |
| 10MWT | -0.43 | -0.95 | -1.2 | -0.37 (-2.18, 1.44) |
