# Supplementary figures and images for: Gait rehabilitation for foot and ankle impairments in early rheumatoid arthritis: a feasibility study of a new gait rehabilitation programme (GREAT Strides)
Source: Pilot Feasibility Stud. 2022 May 30;8:115. doi: 10.1186/s40814-022-01061-9 (PMC9150324; doi:10.1186/s40814-022-01061-9)

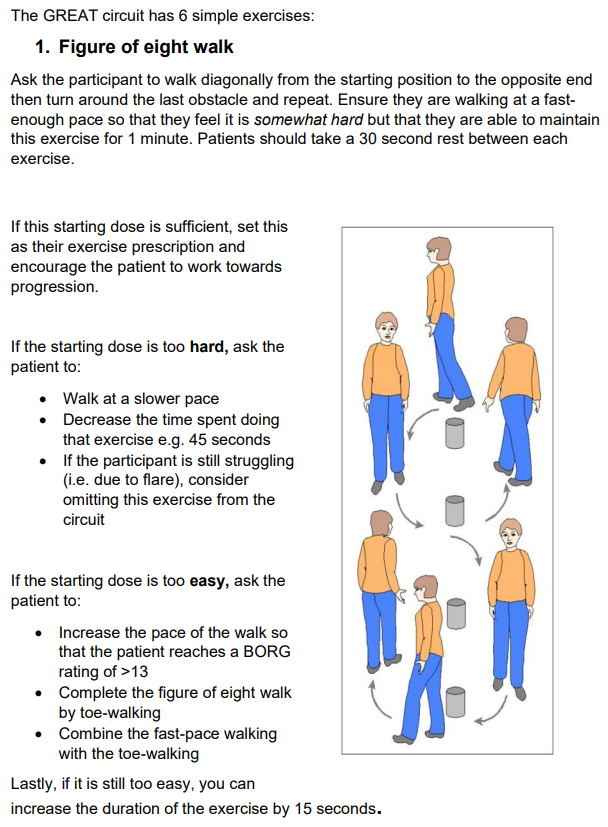


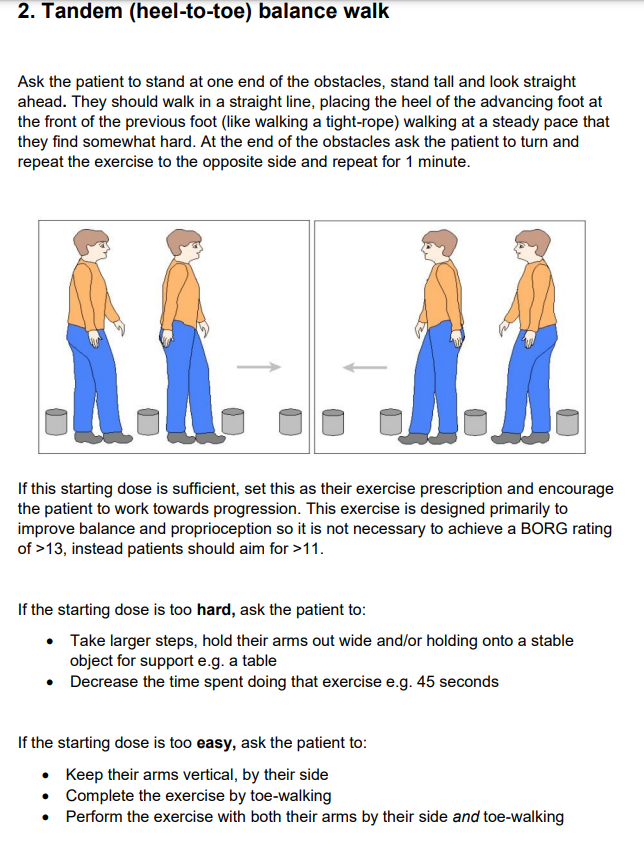


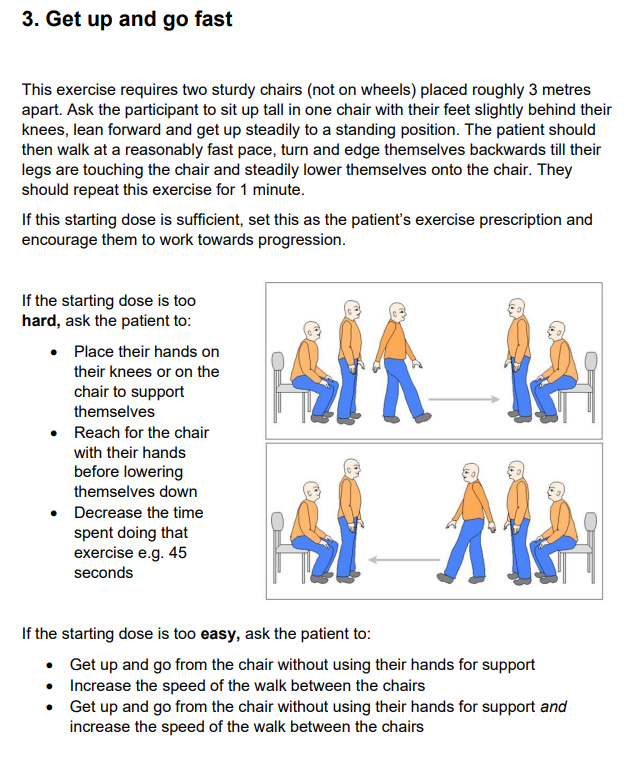


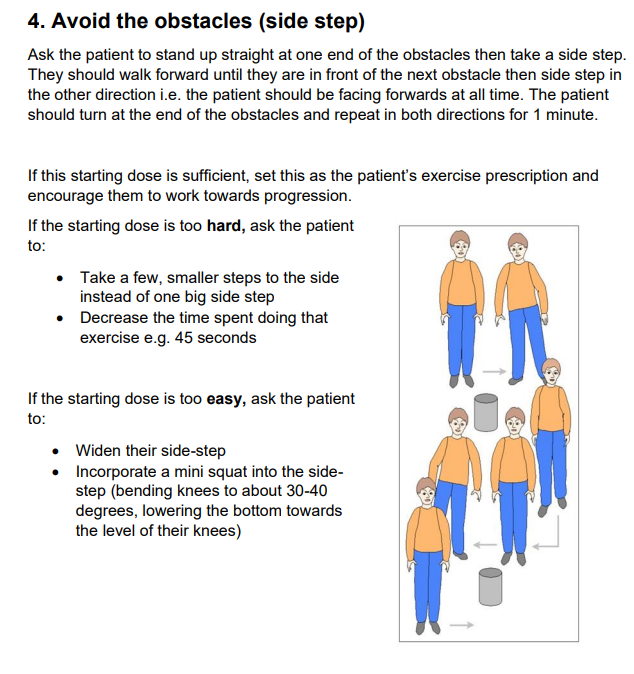


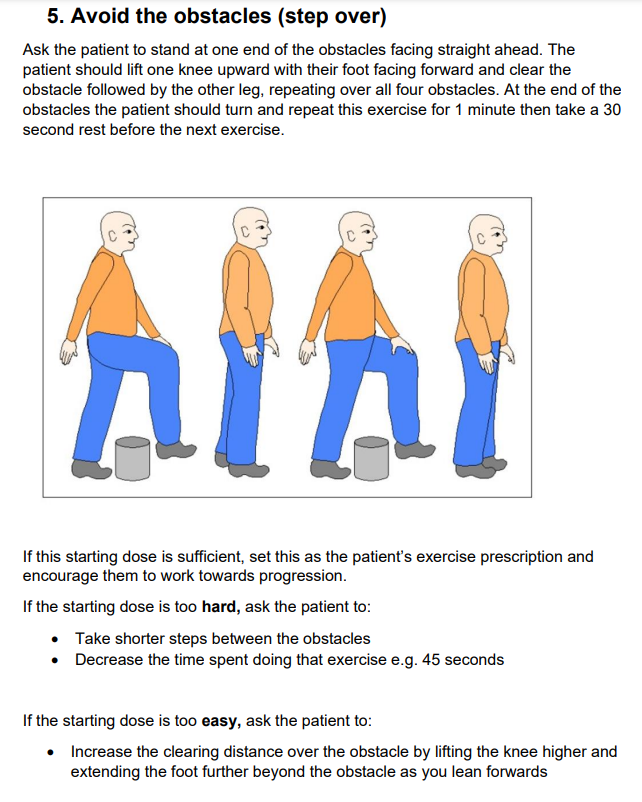


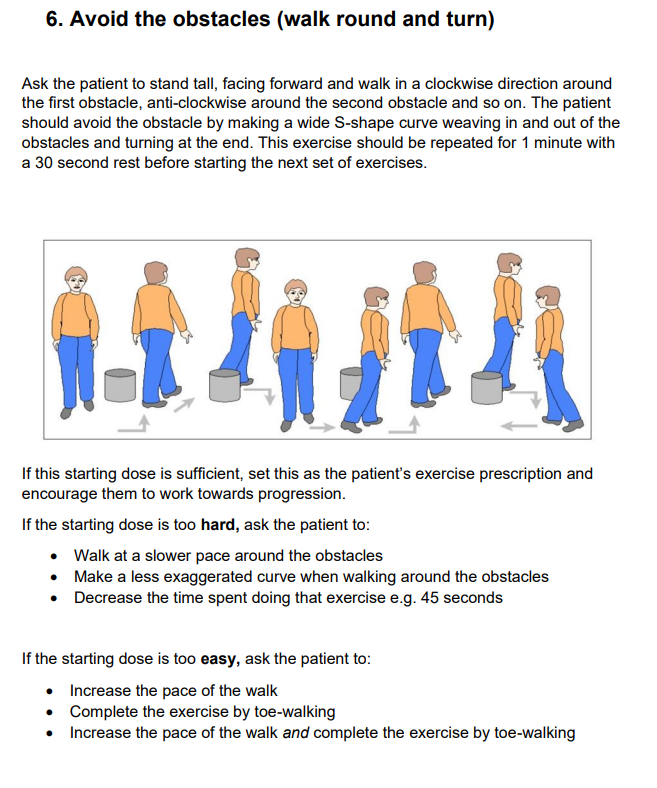


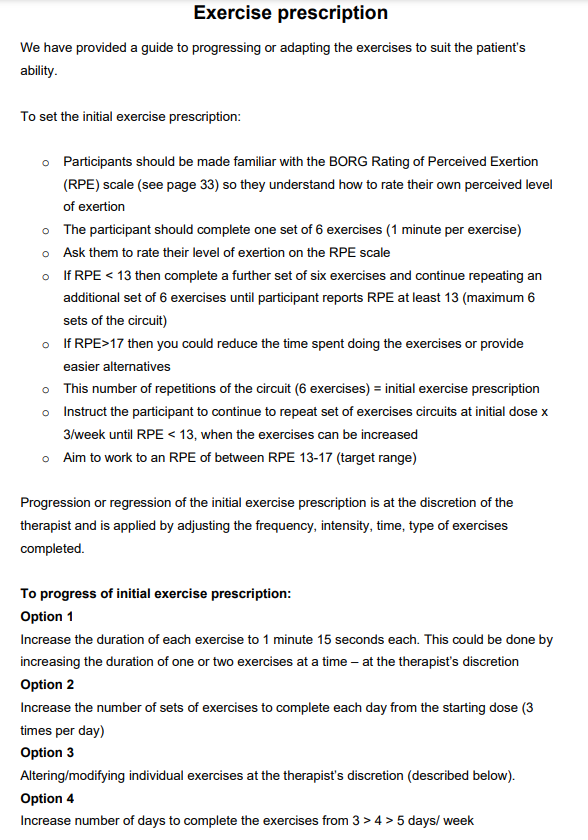


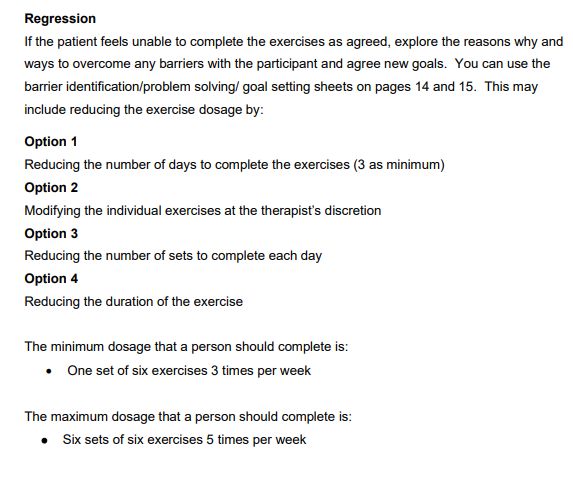

Supplement: Supplementary file 6 — Additional file 6. [file 40814_2022_1061_MOESM6_ESM.docx]
